# Counts of mechanical, external configurations compared to computational, internal configurations in natural and artificial systems

**DOI:** 10.1371/journal.pone.0215671

**Published:** 2019-05-08

**Authors:** Amy LaViers

**Affiliations:** Mechanical Science and Engineering, University of Illinois at Urbana-Champaign, Urbana, IL, United States of America; Pavol Jozef Safarik University in Kosice, SLOVAKIA

## Abstract

Animal movement encodes information that is meaningfully interpreted by natural counterparts. This is a behavior that roboticists are trying to replicate in artificial systems but that is not well understood even in natural systems. This paper presents a count on the cardinality of a discretized posture space—an aspect of *expressivity*—of articulated platforms. The paper uses an information-theoretic measure, Shannon entropy, to create observations analogous to Moore’s Law, providing a measure that complements traditional measures of the capacity of robots. This analysis, applied to a variety of natural and artificial systems, shows trends in increasing capacity in both internal and external complexity for natural systems while artificial, robotic systems have increased significantly in the capacity of computational (internal) states but remained more or less constant in mechanical (external) state capacity. The quantitative measure proposed in this paper provides an additional lens through which to compare natural and artificial systems.

## Introduction

Moving bodies seem to express information: animals, including humans, communicate through non-verbal, visual cues. For example, a nervous twitch of the eye, slight straightening of the spine, or subtle quickening of breath of a poker player may involuntarily occur because they have just drawn a fortuitous card, reflecting a change of their internal estimate of their likelihood to win the hand. Another player may, having watched this opponent over the course of several hands, recognize this “tell” and adjust their own strategy accordingly. Or, perhaps the first player is bluffing, and the action of the eye, spine, and breath are created to throw off their opponents. In either case, information is being transmitted and received. Inside the context of a poker game, these changes in external presentation have meaning.

One small part of the example described in the previous paragraph is the capacity for humans to transmit information through their bodies. Motion over time can transmit arbitrary amounts of information (consider an LED using Morse Code to encode information), but the expressiveness of instantaneous postures may be an important practical measure of information sharing (consider an array of LEDs versus the single LED). Such a measure would not quantify information obtained from velocity of change in posture, which is an important potential source of information. Moreover, any metric on source complexity does not capture the effect of a source on a receiver in context (consider an array of LEDs displaying information in a language unfamiliar to the human receiver).

This paper uses a traditional, information-theoretic communication model, relating each element of the example to either a source (the player who draws a card), channel (the poker game), or receiver (the other players at the table) [1]. Inside this picture, this paper deals only with establishing order-of-magnitude bounds on the practical capacity of a mechanical system as an information *source*. Prior work has used approximate entropy as a measure of system complexity [2], and more recently, symplectic principle component analysis and Shannon entropy have been used to characterized complex systems, such as time series data of electroencephalograms of human brains and vibrations from mechanical systems [3]. This paper will take a bottom-up modeling approach, counting the number of configurations available to systems and using Shannon entropy to measure posture spaces that are discretized according to the resolution of sensing architecture.

Animal posture and movement encode information that is meaningfully interpreted by natural counterparts [4]. Coordination in groups [5], social interactions [6], and cuing during physical collaboration [7] are tasks that autonomous agents are being designed to replace or supplement natural counterparts where information about internal state of the system needs to be expressed through a physical body. Fish schooling [8, 9] and starling flocks [10] (to name a few) have been studied through the lens of dynamical systems theory and related to human crowd behavior [11]; in each case, agents take local cues from nearby neighbors to produce complex global behavior. Similarly, in dyadic interactions between humans, mirroring movements facilitate communication transfer [12]. These are examples where phenomena modeled by *continuous* dynamic equations facilitate motion coordination. But, at the same time, *discrete* information transfer is occurring. Evidence shows that behavior complexity increases with encephalization quotient across a sampling of species [13], and this type of trend analysis by species has been shown to correlate number of distinct behaviors observed with number of distinct muscle types [14].

Another way of asking these questions is through lens of imitation. The success of this goal is often determined through a functional lens, where one aspect of behavior of a system component, e.g., a specific joint angle behavior over time, is faithfully recreated. The review in [15] discusses “robots that imitate humans”, describing “very high fidelity playback.” This notion of imitation refers to matching the value of specific joint angles of each system over time. This established route of imitation through trajectory-based comparison of data capture is used by many researchers, comparing to human counterparts [16] and other animals [17–19], with particular attention to the precision of end points of appendages [20] or rate of activity [21]. In [22–27] motion capture data, a sparse representation, using a 10s or 100s of degrees of freedom, of human movement, seeds artificial system behavior. In this vein, even very simple (2 degree-of freedom) robots have been described by humans as “imitating” human motion capture data when shown correlated behavior over time [28] On the other hand, models in animation are orders of magnitude more complex, using 1,000s and 10,000s of parameters [29–31]. That is, researchers do not have a clear agreement about the order of magnitude of the number of distinct postures available to humans or whether that complexity aids agility. Moreover, features measured through complexity may better align with the trends identified in the uncanny effect of some human-like machines [32].

Optimization has been proposed to describe natural motion for decades. The minimum jerk model [33] has been extended over time with many variants [34], including one that leverages stochasticity [35]. In that work, Todorov provides a model that reconciles human complexity with human performance in complex environments, arguing that humans have many more degrees of freedom than they need but that this makes humans robust to failures in dynamic environments [36]. In factory settings, robots have been out-performing humans for decades, offering greater precision, higher-payloads, and consistency in repeatable tasks [37–39]. Imitating the movement of biological organisms, using similar, trajectory-matching metrics for success, has been a topic in animation [40] and robotics [41, 42]. From the point of view of Newton and mechanics, robots are superior mechanical devices. Yet, despite an explosion of computing power, including cloud-based devices, robots do not thrive in dynamic environments nor can they recreate the social behaviors of humans, even under teleoperation [43]. In other words, by the continuous, mechanical models of dynamical systems where the integral relationships between position, velocity, and force are demonstrated, robots are outperforming humans, but in practice robots are not rapidly replacing humans in complex environments.

The idea that communication through movement may require behavior that counters efficiency has been demonstrated through the comparison of two defined ideas “predictability” and “legibility” in [44]. This work provides a clear model for how efficiency and clarity of intent may be at odds, but considers only simple goal transmission through movement, distinguishing between two objects: this bottle or that. In poker, there are 10 different categories of hands, e.g., a flush, a full house, with about 2.5 million hands possible. In other words, there is likely more bandwidth than one bit in the channel between humans engaged in this activity. Previous work has explored how much information is needed in tasks of *sensing* the environment for artificial systems [45, 46] and natural ones [47]. Many of these same trajectory-based, continuous joint angle measures have been applied in social contexts where varied messages are created through modulation of movement design. Human actors provide motion trajectories for “expressive” or “affective” movement in mobile robotics [48], articulated robots [49], and gait animation [50]. These efforts, which showcase the power of work by artists, show it’s possible to communicate through artificial systems.

However, the capacity to communicate through machines is noticeably limited. Indeed, in successful examples, like movies, performances, and other artistic work, the context around these machines (real or simulated) must be highly controlled. Prior work shows that even a motion model derived from human motion (and not as yet implementable on machines) expresses very different ideas when exposed to and situated in variable environmental contexts [51]. It’s been shown that people read narrative even into only “apparent” behavior [52]. But, in practice work, artists have found that this is especially true for robotic motion [53], which has, in some way, less specificity than that of human counterparts. This paper provides a quantitative measure that may explain this observation and may help understand a key difference between natural and current artificial systems.

## Materials and methods

To begin, consider some established examples of information sources:

- A simple LED. It is always in one of 2 states. By flashing on and off, it can communicate one bit of information. Using a coding scheme, e.g., Morse code, this system can transmit an arbitrary amount of information given an infinite time horizon.
- A trio of three LEDs. It is always in one of 2^3^ = 8 states. By flashing each LED on and off in turn, it can communicate three bits of information. We might say that adding two LEDs to the previous system created a more expressive system.
- A large array of *n* LEDs. It is at any given time in one of 2^*n*^ states. By turning on and off elements in this array, more complex messages, of size *n* bits, can be transmitted.

Given an infinite time window, these systems can all communicate arbitrary amounts of information. What distinguishes their level of practical usefulness is the number of externally visible configurations accessible to the system. Unsurprisingly, computer monitors are essentially comprised of many lighted elements, roughly equivalent to LEDs, whereas the on/off indicator on a coffee pot may only be a single LED. This reflects the fact that the complexity of the internal state that each of these systems need to communicate to the environment is different. The discrete measure listed above, quantifying the capacity of LED *sources*, has limitations:

- Potential correlations in on/off patterns may result in compressibility of the source behavior, e.g., if all LEDs fire on together all of the time a sparser representation is possible.
- Dynamic changes, e.g., a notably slow versus quick onset of the “on” state, can also add to source complexity and are not captured.

When considering information transfer to humans, important limitations of these *receivers* should be enumerated, as they come into play for mechanical systems too:

- The timescale of the source changes may be too fast for the human to perceive.
- The brightness of the on state may not offer enough contrast for the human to perceive.
- The size of the individual LEDs may be too small for the human to perceive.
- The human receiver may not know Morse code (or whichever encoding scheme is used).

Note that these limitations are not homogeneously distributed in the population. Some humans know Morse Code; some do not. Some have more sensitive eyes than others. Moreover, if the culture or upbringing of the human receiver is different from the one who encoded the message in the first place, the meaning of the message may be lost. There are plenty of schemes, like grouping the action of LEDs to provide the correct resolution or using an intuitive encoding that will improve the performance of the system in transmitting information across a *channel* to a *receiver*.

Thus, to quantify the complexity of this proposed *source*, let *N* be the number of actuator *types* on a machine. Let *M*_*i*_ be the number of degrees of freedom with a particular number of available configurations *R*_*i*_, which is computed via counting from an actuators minimum to maximum range via its resolution. From this description of a machine’s construction,

$$\begin{array}{c} C=\prod_{i=1}^{N}R_{i}^{M_{i}} \end{array}$$ (1)

is the number of discrete, kinematic configurations (shapes) available to a platform. From there one measure of the capacity of that platform is the amount of information, or Shannon entropy, which is measured in *bits*, needed to uniquely identify a configuration for that platform. This is given by

$$\begin{array}{c} K=\log_{2}\left(C\right). \end{array}$$ (2)

On an array of *n* LEDs (or *n* transistors), this equation becomes

$$\begin{array}{c} K=\log_{2}\left(\prod_{i=1}^{1}2^{n}\right)=n. \end{array}$$ (3)

On a robot with a simple gripper (which is either open or closed), two identical servos, and a single LED *N* = 3. Assume each servo has 360° range and is sensed by an encoder with 0.1° resolution with a gripper that may be ‘open’ or ‘closed’ and an LED that may be ‘on’ or ‘off’, *R*_1_ = 3600 with *M*_1_ = 2 and *R*_2_ = 2 with *M*_2_ = 2. This becomes

$$\begin{array}{c} 2^{2}\times 3600^{2}=5.2\times 10^{7}\;\text{configurations.} \end{array}$$

This means the robot, as an information source, can express

$$\begin{array}{c} \log_{2}\left(5.2\times 10^{7}\;\text{configurations.}\right)\approx 26\;\text{bits} \end{array}$$

of information, through a static, instantaneous configuration, in its environment. Moreover, for this robot, most of its complexity comes from *mechanical* actuators. Removing the LED from the analysis gives

$$\begin{array}{c} \log_{2}\left(2^{1}\times 3600^{2}=2.6\times 10^{7}\;\text{configurations}\right)\approx 25\;\text{bits}. \end{array}$$

Thus, this simplistic robot can be compared to a 25-bit display in terms of the cardinality of a discretized space of possible mechanical configurations, which is limited in a meaningful way by the resolution of sensing, actuation, and control subsystems.

Any computer that is Turing-complete can, in theory, run the same programs as other complete machines. However, the number of transistors in the CPU is a useful, implementation-specific measure (which has been growing) to understand how practically powerful a given machine is, despite the static nature of this counting. After two detailed examples are presented, a similar analysis will quantify patterns observed through using robots in dance performances mentioned in the Introduction.

### Example: SoftBank/Aldebaran NAO humanoid robot

Table 1 outline the basic capabilities of the NAO [54–56] where the sensor resolution (an encoder with 0.1° precision, which corresponds to the model specs [57]) has been used to determine *R*_*i*_.

**Table 1:** Robot system details. NAO robot degrees of freedom description [55].

**Mechanical DOF**	**Range / Resolution**	*R*_*i*_
l/r hand	open/close	2
head yaw	-119.5 to 119.5 / .1	2390
head pitch (at 0 yaw)	-38.5 to 29.5 / .1	680
l/r shoulder pitch	-119.5 to 119.5 / .1	2390
l/r shoulder yaw	-119.5 to 119.5 / .1	2390
l/r shoulder roll	-88.5 to -2 / .1	865
l/r wrist yaw	-104.5 to 104.5 / .1	2090
pelvis	-65.6 to 42 / .1	1076
l/r hip roll	-21.7 to 45.2 / .1	669
l/r hip pitch	-88 to 27.7 / .1	1157
l/r knee pitch	-5.3 to 121.0 / .1	1263
l/r ankle pitch	-68.2 to 52.9 / .1	1211
l/r ankle roll	-22.8 to 44.1 / .1	669
**Other DOF**	**Range / Resolution**	*R*_*i*_
eye LEDs (16)	off or one of 12 colors	13
ear LEDs (20)	off or one of 12 colors	13
foot LEDs (2)	on/off	2

Thus, the complexity of external change is calculated as

$$\begin{array}{c} K=\log_{2}(2^{2}\times 2390^{1}\times 680^{1}\times 2390^{2}\times 2390^{2}\times 865^{2}\times 2090^{2}\times 1076^{1}\ldots \\ \times 669^{2}\times 1157^{2}\times 1263^{2}\times 1211^{2}\times 669^{2}\times 13^{16}\times 13^{20}\times 2^{2})\ldots \\ =\log_{2}\left(2.4\times 10^{106}\;\text{configurations}\right)\approx 353\;\text{bits} \end{array}$$ (4)

Removing the LED systems, the capacity of mechanical postures is calculated as

$$\begin{array}{cc} K & =\log_{2}\left(2^{2}\times 2390^{1}\times 680^{1}\ldots \times 1211^{2}\times 669^{2}\right)\\ & =\log_{2}\left(4.7\times 10^{65}\;\text{configurations}\right)\approx 218\;\text{bits} \end{array}$$ (5)

This calculation includes physical combinations which are kinematically or dynamically infeasible, which would reduce this calculated complexity. The capacity for changes in motor speed between configurations is not reflected here and would increase the complexity of behavior. Thus, the number may be seen as a measure analogous to a transistor count on an integrated circuit.

### Example: Bellagio water fountains

Consider a tourist attraction, like the Bellagio water fountains in Las Vegas, NV. Tourists line up every hour to watch this famous display, routinely included in lists of popular Vegas attractions. This is to say that the fountain display is visually very interesting for human watchers; therefore, it should be more expressive than the NAO robot. The fountain has about 1,200 water cannons with 5,000 lights as part of its display. It also has the ability to create fog and features popular music during the shows. For this analysis [58, 59], consider only the water cannons and lights. The cannons come in four types: robotic Oarsman and three sizes of Shooters. The 208 Oarsman are articulated cannons with active control; the Shooters simply blast water at three predetermined pressure settings, each having a single pressure setting according to their size. The lights can be a range of colors.

Table 2 articulates a model for this system. For the Oarsman, which rotate about two axes, assume a range of motion of 160° with a resolution of 1° in each dimension. Assume the water shooting out of the cannon to be on or off with a single pressure setting. Likewise, the Shooters, are either on or off without articulation. The lights can be ‘off’ or one of twelve colors (as modeled by a moderate segmentation of the color wheel). The following analysis will ignore the music that plays alongside.

**Table 2:** Fountain system details. Estimated fountain system degrees of freedom [58, 59].

**Mechanical DOF**	**Range / Resolution**	*R*_*i*_
Oarsmen RX (208)	0° to 160° / by 1°	160
Oarsmen RY (208)	0° to 160° / by 1°	160
Oarsmen water (208)	on/off	2
Shooters (1,175)	on/off	2
**Other DOF**	**Range / Resolution**	*R*_*i*_
lights (6,200)	off or one of 12 colors	13

Computing the instantaneous mechanical complexity, ignoring the lights, we find the following:

$$\begin{array}{c} K=\log_{2}\left(2^{1175+208}\times 160^{208+208}\right)=\log_{2}\left(1.7\times 10^{1333}\;\text{configurations}\right)\approx 4,429\;\text{bits.} \end{array}$$ (6)

Including lighted degrees of freedom, we find the following considerable increase in expressivity:

$$\begin{array}{c} K=\log_{2}\left(2^{1175+208}\times 160^{208+208}\times 13^{6200}\right)=\log_{2}\left(4.9\times 10^{8239}\;\text{configurations}\right)\approx 27,372\;\text{bits.} \end{array}$$ (7)

Thus, the proposed metric provides one possible quantitative bound on *how much more expressive* the fountains are than the prior humanoid example. In this case, about two orders of magnitude with respect to the amount of information they can encode. This result is consistent with the considerably great system expense and tourist attendance of the fountains; however, any analysis of the physical characteristics of either system cannot explain the interest on the part of humans in either system. The city of Las Vegas, inclusion in movies, and other cultural factors play a large role in the *context* surrounding these expressive fountains. Music and the sound of water spray, which also accompanies the fountain displays, also adds an audible degree of freedom while the feel of rushing air adds a haptic channel that adds to the experience. On the other hand, it may be that the human-like form of the NAO robot makes it particularly interesting to human observers.

This measure merely quantifies one small aspect of the myriad of factors that create entertainment value and meaningful motion for human observers. Moreover, as in the previous example, we do not capture the additional expressivity that the dynamics of timing and water add to (and take away from) the system. For example, by moving at variable speeds, these fountains can create different patterns in the water, which add to the system’s complexity. On the other hand, in the presence of water, not all points in the cannon’s range might be physically feasible.

What if all the water cannons were the articulated, Oarsman variety? In that case, we have:

$$\begin{array}{c} K=\log_{2}\left(2^{1383}\times 160^{1383}\times 13^{6200}\right)=\log_{2}\left(1.2\times 10^{10371}\;\text{configurations}\right)\approx 34,452\;\text{bits}. \end{array}$$ (8)

If, in addition, we boost the resolution of each cannon of the original system to 0.1°, we have:

$$\begin{array}{c} K=\log_{2}\left(2^{1383}\times 1600^{1383}\times 13^{6200}\right)=\log_{2}\left(1.2\times 10^{11754}\;\text{configurations}\right)\approx 39,046\;\text{bits}. \end{array}$$ (9)

Thus here, we can see how adding water cannons and articulation resolution increases the expressivity of the platform, but does not change the order of magnitude of K. The next section formalizes this with sensitivity analysis by incorporating two uncertainty parameters into the model.

### Sensitivity analysis

In this analysis *M*_*i*_ (note ∑_*i*_ *M*_*i*_ = *N*) and *R*_*i*_ are platform-specific parameters that may involve estimation, particularly for natural systems, and are sources for model error. This section considers the sensitivity of the model to two sources of uncertainty: number of degrees of freedom and precision of each degree of freedom. Let *η*_*i*_ be the uncertainty associated with the number of degrees of freedom of a particular type, *M*_*i*_. If the number is too low, *η*_*i*_ should be added to *M*_*i*_ and if *M*_*i*_ is an overestimate, *η*_*i*_ should be subtracted. Let *ϵ*_*i*_ be the uncertainty associated with the precision of *R*_*i*_. If the precision used to generate *R*_*i*_ is too coarse, then this factor needs to divide *R*_*i*_ while if it is too fine, then this factor needs to multiply *R*_*i*_. These parameters can be incorporated into the calculation, giving a bound on the accuracy of the true entropy $\bar{K}$:

$$\log_{2}\left(\prod_{i=1}^{N}R_{i}/\epsilon_{i}^{M_{i}-\eta_{i}}\right)\leq \bar{K}\leq \log_{2}\left(\prod_{i=1}^{N}R_{i}\epsilon_{i}^{M_{i}+\eta_{i}}\right)$$ (10)

where *ϵ*_*i*_ ≥ 1 and *η*_*i*_ ≥ 0 and if *ϵ*_*i*_ = 1 and *η*_*i*_ = 0, this corresponds to zero uncertainty, returning Eq 3.

To check the sensitivity of this model to each source of uncertainty note the following algebraic expansion of the upper bound on $\bar{K}$:

$$\begin{array}{c} \bar{K}\leq M_{1}\log_{2}R_{1}+\eta_{1}\log_{2}R_{1}+M_{1}\log_{2}\epsilon_{1}+\eta_{1}\log_{2}\epsilon_{1}+\ldots \\ \ldots +M_{N}\log_{2}R_{N}+\eta_{N}\log_{2}R_{N}+M_{N}\log_{2}\epsilon_{N}+\eta_{N}\log_{2}\epsilon_{N}\\ \;\Rightarrow \bar{K}\leq K+\sum_{i=1}^{N}\left[\eta_{i}\left(\log_{2}R_{i}+\log_{2}\epsilon_{i}\right)+M_{i}\left(\log_{2}\epsilon_{i}\right)\right]. \end{array}$$ (11)

For the lower bound, we have:

$$\begin{array}{c} \bar{K}\geq M_{1}\log_{2}R_{1}-\eta_{1}\log_{2}R_{1}-M_{1}\log_{2}1/\epsilon_{1}-\eta_{1}\log_{2}\epsilon_{1}+\ldots \\ \ldots +M_{N}\log_{2}R_{N}-\eta_{N}\log_{2}R_{N}-M_{N}\log_{2}1/\epsilon_{N}-\eta_{N}\log_{2}\epsilon_{N}\\ \Rightarrow \bar{K}\geq K-\sum_{i=1}^{N}\left[\eta_{i}\left(\log_{2}R_{i}+\log_{2}\epsilon_{i}\right)-M_{i}\left(\log_{2}\epsilon_{i}\right)\right]. \end{array}$$ (12)

Thus, error on $\bar{K}$ scales linearly with *η_i_* (degrees of freedom) and *logarithmically* with *ϵ_i_* (precision).

### Limitations of the proposed measure

As with the LED examples previously discussed, the measure proposed here has limitations:

- Potential correlations in exhibited motion may result in compressibility of the source behavior.
- Dynamic changes can also add to source complexity and are not captured.

Moreover, we can acknowledge a similar set of limitations when considering information transfer to humans:

- The timescale of the mechanical changes may be too fast for the human to perceive.
- The change in kinematic position may not offer enough contrast for the human to perceive.
- The size of the changing mechanism may be too small for the human to perceive.
- The human receiver may not know the encoding scheme used by the source, in particular, anthropomorphic systems may have an advantage; this point includes cultural context (which we could consider a type of encoding scheme) as described in the case of the famous Las Vegas landmark, the Bellagio Fountains.

The proposed measure categorizes the instantaneous complexity of a mechanical source, as has been useful in communication theory, ignoring the issues of the channel and receiver. For example, some contextual situations require very simple messages for meaningful interaction, say, answering “this or that” or “yes or no”. Further, human-imitating systems may be more effective in communication to humans. Nor should systems that communicate with humans need to be limited to human shapes—as there are many examples in nature and animated films where non-anthropomorphic characters are quite expressive: we communicate well with dogs and characters of many fictitious forms, for which encoding schemes are learned through contextual interaction and narrative.

## Results and discussion

The measure and procedure presented in the previous section can categorize and compare artificial systems. The results of applying this measure to a variety of robotic platforms and natural systems is presented here. Limitations and assumptions in the analysis are presented as well as discussion of trends in the data.

### Analysis of artificial systems

**Fig 1:**
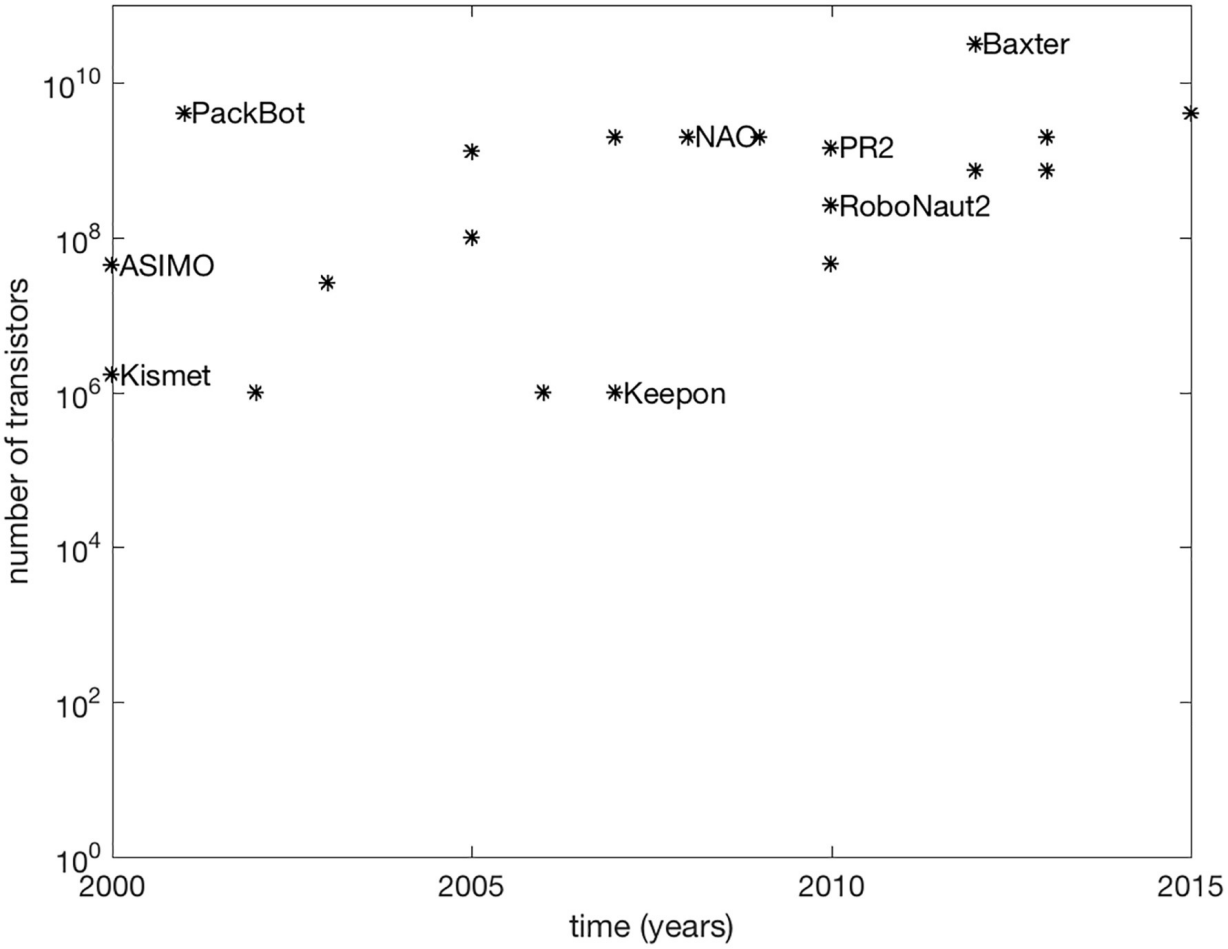
Internal complexity in artificial systems over time. The number of transistors, *t*, used in onboard processors of robots over the last fifteen years. The number of internal configurations available is then 2^*t*^. Some platform names are omitted for clarity; see S1 Appendix for full list.

The plot in Fig 1 shows the number of transistors in the onboard CPU of a range of robots over the past 15 years. While, as many platforms are now Internet connected, it is a limiting picture of the computational power available to these platforms, plots like this have been used to track the progress of computational power over time, which has roughly doubled every year, even serving as a driving goal for the industry (Moore’s Law [60]). In such plots each additional component on an integrated circuit represents the ability to represent a larger—or more precise—number on a single chip. Each new transistor adds a new power of 2 in representation precision.

Note, that the *the number of transistors* in modern, stand-alone processors is in the billions. To convert that to *possible machine configurations*, where the actuators are transistors, the number 2 (which is the number of configurations for each actuator) has to be raised to that large number, resulting in a number of configurations that is bounded by $2^{10^{11}}$ or 10^30102999566^. This number is an important, practical measure of how many numbers the computers onboard robots can represent—or how expressive these internal systems are. Processor speeds and operating system design govern the dynamics of these systems and whether this baseline capacity is used well—counting transistors does not account for this.

**Fig 2:**
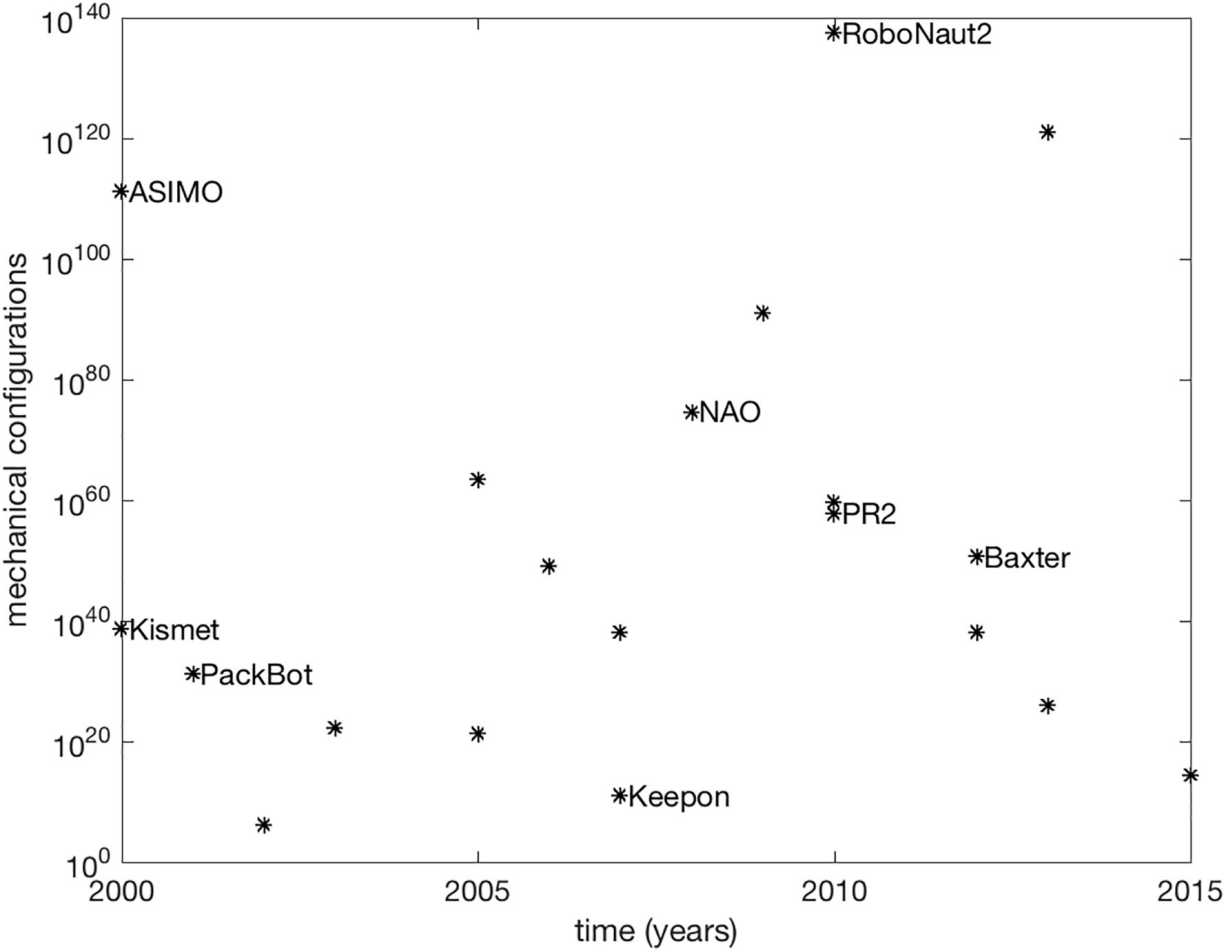
External complexity in artificial systems over time. The number of external configurations available, C, for mechanization in robot platforms over time. Several platforms have an assumed positioning resolution of 0.1° and an estimated range of motion. Some platform names are omitted for clarity; see S1 Appendix for full list.

Fig 2 shows how this measure has evolved on the mechanical configurations of robots over time. The plot shows the number of possible kinematic configurations, C, for a number of rigid-body robots whose motion is governed by motors and encoders measuring their position over time. The degrees of freedom of each robot can be estimated from publications, videos, and online reports; in several cases, the precision of onboard sensors, e.g., rotary encoders in joints, has been assumed to be 0.1 units as in [57, 61, 62] (see S1 Appendix for further discussion and full list of parameters). According to the estimates used here, the corresponding practical measure of how externally complex these robots are is bounded by 10^140^. Like Moore’s proxy of the number of transistors within a single chip, this discretized configuration space does not account for dynamics but gives a starting point for measurement and comparison of instantaneous postural complexity.

**Fig 3:**
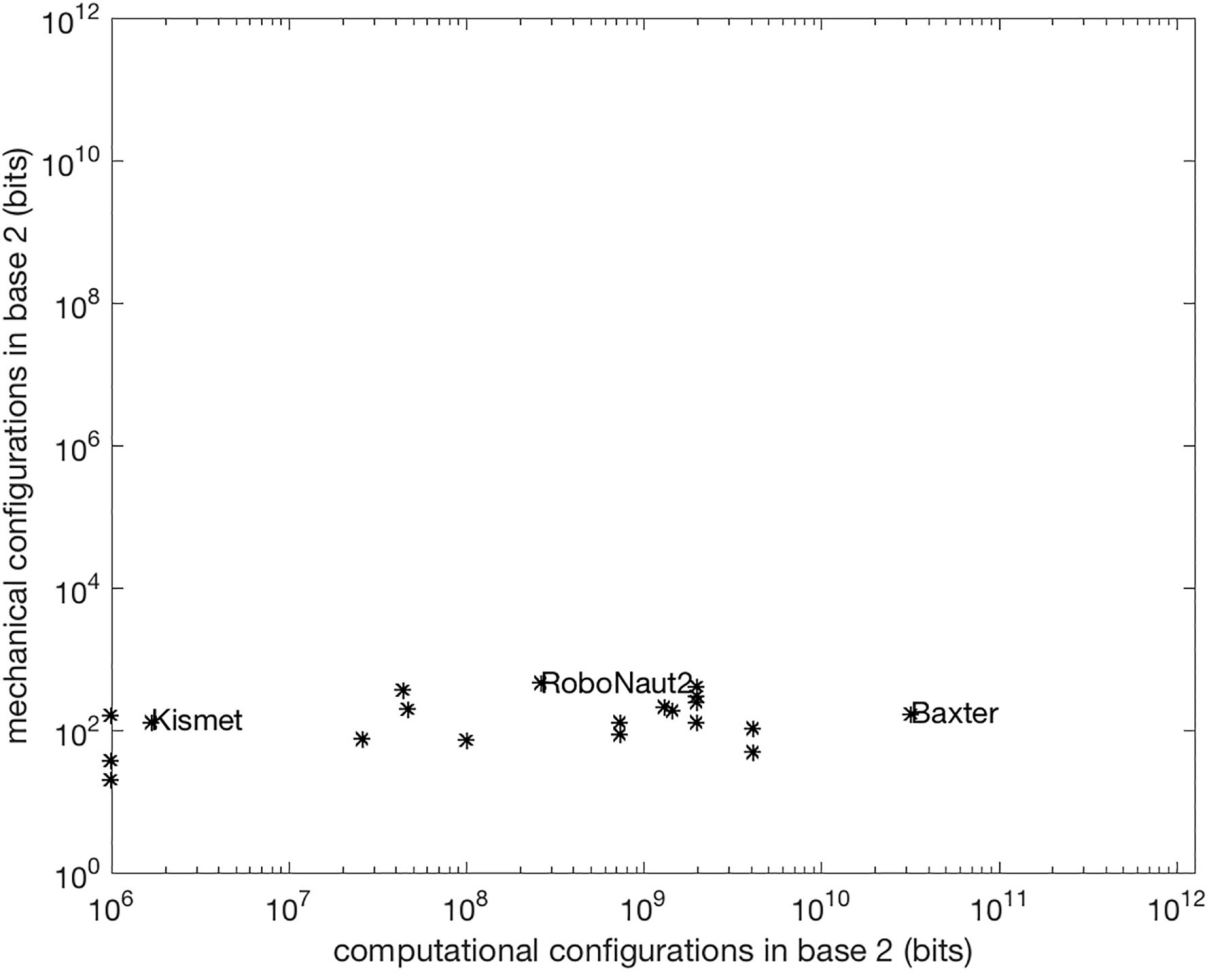
Internal Vs. external complexity in artificial systems over time. A comparison of computational complexity (# of transistors in the CPU) relative to a measure of mechanical complexity (K) on robotic platforms over time. Platform names are omitted for clarity; see S1 Appendix for full list. The unit of measure on both axes is *bits*; square plot axes highlight imbalance between internal and external states (although note that the axes use different ranges).

By converting the number of configurations C to a number represented in a base 2 number system, K, the change in the computational capacities of these platforms can be compared to their mechanization capacities as shown in Fig 3. This log-log plot provides a comparison in terms of the number of bits which it would take to describe the largest number that would fit in the onboard CPU versus the number of bits needed to uniquely represent each pose. The plot reveals an explosion of internal complexity and a flat trend in the mechanical, external complexity over the past 15 years.

The complexity analysis used in the plot shows an imbalance between computation and mechanization capacities. Through this lens, the external complexity of the NAO, the small humanoid robot examined in the prior section, is comparable to the internal complexity a 1960s computer chip with only 256 transistors. This analysis suggests that there could be a magnitude of external complexity that going beyond does not improve system performance or produces intractably complex systems. The next section will attempt to look at natural systems to test this idea further.

### Analysis of natural systems

The previous section outlines a way to compare robot capacity for complex behavior, but the same method can be applied to biological creatures—with the caveat that such systems may involve processes not extant in today’s machines. For example, the difference between sensing and actuation blurs for these systems where movement is tightly linked to sensing, e.g. fovea, and complex, compliant materials comprise much of these systems, e.g., cartilage. However, we can loosely compare “computation” to internal state changes, which do not meaningfully impact the environment, and “mechanization” to external state changes, which cause direct change in the environment.

In prior work, the movement of a microscopic worm (*C. Elegans*) was analyzed using a curve parameterized by 100 angles; then, after capturing an extended period of behavior on laboratory agar, a principle component analysis was performed, revealing that the structure of the behavior could be explained as a superposition of four primary poses [63]. Observing the animal through this lens provided new insights into the behavior of this well-studied animal [64, 65], implying a meaningful parameterization, further explored in [66]. Stephen’s model can be used to compare the *C. Elegans* to modern robots.

A *C. Elegans* has 302 neurons, which can be approximated to be either ‘firing’ or ‘not’ in a static snapshot of time. (This is a commonly used under-approximation, for neurons, which are now believed to use graded, rather than binary, firing mechanisms [67].) Then, each of the 100 angles have a typical range, shown in the empirical results in [63], between −1.5 radians to 1.5 radians, implying 0.1 radians of precision. Thus, using the metric proposed in Eq 3, the kinematic mechanization capacity for this model of a simple animal can be calculated as

$$\begin{array}{c} K=\log_{2}\left(30^{100}\right)=\log_{2}\left(5.2\times 10^{147}\;\text{configurations}\right)\approx 491\;\text{bits}. \end{array}$$ (13)

The linear model derived in [63] can also be used to derive a slightly more compact expression, finding a “compression” that removes correlations in joint angles exhibited. The idea is that the organism, in a particular task or environment, does not utilize the full span of motion modeled by 100 angles. This could be a function of behavioral patterning, environment, or experimental set up; however, the larger behavioral space intoned by the anatomical model may be used in future, unforeseen environments. Here, linear combinations of four “eigenworms” describe 95% of the observed worm behavior. Follow on work estimates this number to be six [66], but the original four-dimensional model is used here.

As an aside, given these lower-dimensional models, it may be easy to then discount the more complex model of behavior entirely, but consider the behavior shown in pavement ants taken to the International Space Station [68]. These ants exhibited unseen behavior for their species in microgravity, suggesting that in new environments animals express new behaviors. In the model proposed here, we suggest that postures exhibited in a particular environment are a compression of possible postures. Namely, without a *rich enough* original high-dimensional space, the lower-dimensional model could not be identified. Thus, both the rich, higher-dimensional model and the sparse, lower-dimensional model derived from observed data will be considered in the analysis to follow.

The weights on each eigenworm, *α*_1_, *α*_2_, *α*_3_, and *α*_4_, also have an observed range, which can be estimated from [63] as *α*_1_: [−2, 2], *α*_2_: [−2, 2], and *α*_3_: [−5, 5]. Four the fourth, which was not found to have behavioral significance and is not provided in [63], assume *α*_4_: [−2, 2]. For each, assume 0.1 in precision based on reported significant figures in [63]. This leads to the following calculation:

$$\begin{array}{c} K=\log_{2}\left(40^{3}\times 100^{1}\right)=\log_{2}\left(6.4\times 10^{6}\;\text{configurations}\right)\approx 23\;\text{bits}. \end{array}$$ (14)

**Fig 4:**
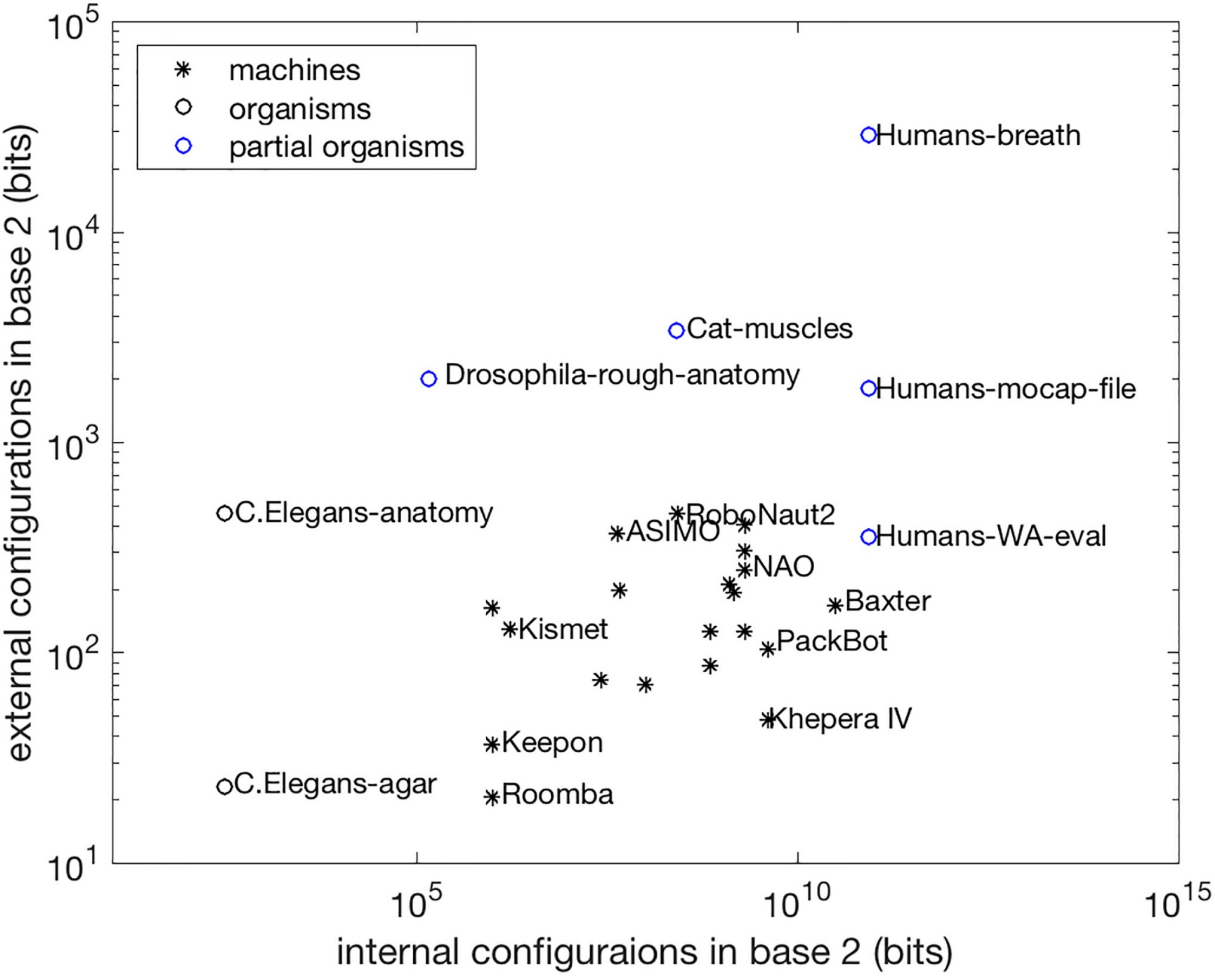
Complexity of natural and artificial systems. The machine data points, plotted in black stars, are the same as those in Figs 1–3. Corresponding points for two models of *C. Elegans* are plotted in black circles. These x-axis points are based on the number of neurons (302); the y-axis data points reflect established behavioral models, one based on anatomy and the other based on exhibited behavior on agar [63]. Other organisms, with less established behavioral models, are plotted in blue circles. The x-axis values for the other organisms are also based on the estimated number of neurons [69]; the y-axis values are given by: Drosophila-rough-anatomy: analysis of [70]; Cat-muscles: generalizing the model of muscles in [71]; Humans-WA-eval: the structure of [72]; Humans-mocap: the structure of a Natural Point OptiTrack motion capture file; and Humans-breath: the simulation in [29]. See S1 Appendix for data detail.

The summary of this analysis is plotted in black in Fig 4. The plot gives a sense of the capacity of today’s robots. It is shown that, through the lens proposed here, *C. Elegans* are apt natural analogs for the mechanical complexity of many robots. While, clearly, *C. Elegans* cannot replicate any of the specific functions that robots accomplish, perhaps they can do equivalently *complex* tasks. That is, for example, painting a particular, single part over and over might also be accomplished by a linear combination of four postures. This is consistent with the fact that today’s robots are created for single-tasks in controlled environments. *C. Elegans* must forage for food and carry out other critical to life tasks in real, dynamic environments. Note that additional factors need also to be considered in this comparison. For example, there are distinct Reynolds numbers at play. Inertial forces dominate the selected robots, while viscous forces dominate the motion of *C. Elegans*; however, this analysis is not considering forces, only the complexity of postural snapshots.

Such detailed motion models for other animals are not common, but a few estimates and examples are included in blue in Fig 4. Relevant work done on fruit flies (*Drosophila*) abstracts their state as a vector with a heading over time [73], but an anatomical analysis via [70] can estimate postural complexity of the organism. Researchers look to muscle activity involved in postural control for cats with weights having precision to the hundredth decimal place [71]; generalizing this precision to all estimated 517 [74] muscles in cats gives an estimate of the complexity of their muscular control, leaving out the mechanical advantages of their compliant bones, skin, and hair. For human motion, three different established guides to behavior (a public health diagnostic [72], a Natural Point OptiTrak motion capture file, and an animation of breathing [29]) of body posture can be used to show similarity between these models and those that identified behavioral understanding of *C. Elegans*.

The balance between internal and external capacity seen in *C. Elegans* is not found in robots or models of other organisms. This could be due to a lack of benefit in increased external complexity, suggesting that there may be a natural threshold on order-of-magnitude complexity, around which systems cluster. However, the discrepancy between human motion models suggest that we may not yet appreciate the role of complexity in natural motion (and whether it is needed to describe the Shannon entropy of these systems).

## Conclusion

The paper has introduced a measure for postural expressivity, clarifying prior points of view on function versus expression in movement by relating these two ideas in the same measure. Finally, the paper uses this proposed measure to compare extant artificial systems to natural systems, suggesting that, through the lens of complexity, many modern robots, including humanoids, are comparable to a microscopic worm. This provides a quantitative model for ways in which robots still have to improve to recreate the behavior of natural counterparts (without negating the fact that the force and velocity profiles of these machines can certainly exceed that of *C. Elegans* and many natural systems). Future work will extend the discretized configuration space presented here to a discretized state space, according to actuator force and torque limits, in order to account for information transmitted through variable velocity.

Discrete measures may be important in highlighting the differences between natural and artificial systems. For example, an Ising model has been used to describe differences between natural and human-made, urban environments where images of natural scenes versus urban ones are shown to have different statistical properties [75]. To create more characters in a font style, such as emoji, a more complex underlying ASCII representation is needed [76]. This paper uses a similar approach to begin to characterize the ability for systems to transmit information through their mechanical motion. This may begin to bring to bear a notion of expressivity in robotics that is consistent with usage in computer science [77], genetics [78], psychology [79], and dance. Moreover, this measure may aid in comparisons of soft-bodied, color-changing animals with many appendages like some cephalopods to mammals like baboons, which are discussed in [80] in terms of the expressivity of their behavior.

This work provides evidence relevant to the discussion around the effect of machine-based automation of human tasks, particularly in manufacturing. Humans operate in dynamic environments where variability in movement is essential in accommodating and coping with an unpredictable world. Robots do well—and in many cases are superior—in repeatable tasks where environmental factors are controlled (as in a factory), but do not have the same capacity to adapt or function in complex, variable environments. The trends pointed to here in robotics may help guide system development: in hardware, soft and compliant robots which inherently have more instantaneous complexity due to the rich space of shapes taken on by these systems, and, in software, increasing motion variability will offer robotic systems greater expressivity. In human-robot interaction, quantitative guides on how many possible variable states a given platform can produce may help ensure clarity in user perception. Active, promising work in these areas include compliant mechanisms [81], soft components [82], and modular systems [83]. Further, considering the wide, possibly unused in typical activities, capacity for motion of natural systems, may produce improved understanding of how animals adapt. Just as information has been used to characterize the sensory systems of animals [84], it may prove to be a useful lens through which to study motor behavior.

## Supporting information

S1 Appendix Numbers used to calculate data points in Figs 1–3, including those in Tables 1 and 2, are provided as a download-able dataset. A few notes about the elements of this dataset are provided here:
The list of 20 artificial systems analyzed is Kismet (2000), ASIMO (2000), PackBot (2001), Roomba (2002), RoboSapien (2003), BigDog (2005), KR 60 HA (2005), Aibo (2006), Keepon (2007), LittleDog (2007), NAO (2008), Simon (2009), Darwin (2010), PR2 (2010), RoboNaut2 (2010), Baxter (2012), Cheetah (2012), LBR iiwa (2013), HansonZeno (2013), and Khepera IV (2015).For the external element counts of artificial systems, many of these were determined through observation. The choice of 0.1 resolution, where not directly available, is supported by the use of this precision of encoders in the NAO platform [57], in a patent filed by FANUC Corp. for their industrial robots [61], and in a more recent patents on encoders by Bei Sensors and Systems Company, Inc. [62]. In these systems, encoders use optical sensors to detect light transmission through transparent regions on a circular disc; this analog signal is transmitted to digital representation, where the number of bits allotted can also impact resolution. A common number of transparencies is, as in the FANUC patent, 2500, and a common digital representation is 12 bits as in a more recent patent by Bei, allowing 4096 distinct positions. Across 360° both result in roughly 0.1° precision. In applications like laser jet printers, these devices can also achieve micrometer precision (roughly 1 million positions out of one rotation) as discussed in a patent from Lexmark International, Inc. [85]. To this author’s knowledge, such specialized, high-precision hardware is not common in the robots reviewed here.For the natural systems analyzed, the values used are based on [29, 63, 70–72, 74].
(XLSX)
